# Human ovarian cancer intrinsic mechanisms regulate lymphocyte activation in response to immune checkpoint blockade

**DOI:** 10.1007/s00262-020-02544-5

**Published:** 2020-03-21

**Authors:** Marina Natoli, Nair Bonito, James D. Robinson, Sadaf Ghaem-Maghami, Yumeng Mao

**Affiliations:** 1 Department of Surgery and Cancer, Institute of Reproductive and Developmental Biology, Imperial College London, London, UK; 2 Mechanistic Biology and Profiling, Discovery Sciences, R&D, AstraZeneca, Cambridge, UK; 3 Bioscience, Early Oncology R&D, AstraZeneca, Cambridge, UK; 4 Science for Life Laboratory, Department of Immunology, Genetics and Pathology, Uppsala University, Uppsala, Sweden

**Keywords:** Human ovarian cancer, PD-1/PD-L1 blockade, Immune resistance, Human cancer/immune co-culture

## Abstract

Immune checkpoint blocking antibodies are currently being tested in ovarian cancer (OC) patients and have shown some responses in early clinical trials. However, it remains unclear how human OC cancer cells regulate lymphocyte activation in response to therapy. In this study, we have established and optimised an in vitro tumour-immune co-culture system (TICS), which is specifically designed to quantify the activation of multiple primary human lymphocyte subsets and human cancer cell killing in response to PD-1/L1 blockade. Human OC cell lines and treatment naïve patient ascites show differential effects on lymphocyte activation and respond differently to PD-1 blocking antibody nivolumab in TICS. Using paired OC cell lines established prior to and after chemotherapy relapse, our data reveal that the resistant cells express low levels of HLA and respond poorly to nivolumab, relative to the treatment naïve cells. In accordance, knockdown of IFNγ receptor expression compromises response to nivolumab in the treatment naïve OC cell line, while enhanced HLA expression induced by a DNA methyltransferase inhibitor promotes lymphocyte activation in TICS. Altogether, our results suggest a ‘cross resistance’ model, where the acquired chemotherapy resistance in cancer cells may confer resistance to immune checkpoint blockade therapy through down-regulation of antigen presentation machinery. As such, agents that can restore HLA expression may be a suitable combination partner for immunotherapy in chemotherapy-relapsed human ovarian cancer patients.

**Electronic supplementary material:**

The online version of this article (10.1007/s00262-020-02544-5) contains supplementary material, which is available to authorized users.

## Introduction

Reinvigorating tumour recognition by the human immune system through blockade of the PD-1/PD-L1 axis has demonstrated clinical efficacy in patients with advanced cancers [1]. However, multiple resistance mechanisms induced by the oncogenic pathways can hamper the overall success in a broad range of cancer types [2]. Several predictive biomarkers including PD-L1 expression on cancer cells and tumour mutational burden (TMB) in cancer cells have been proposed to enable disease positioning and patient stratification [3].

Patients with ovarian cancer (OC) are usually diagnosed at an advanced stage. Most patients are treated with surgery or conventional chemotherapeutic agents including carboplatin and paclitaxel without stratification [4]. Although OC patients can respond to the initial chemotherapy, resistance often occurs which leads to a low 5-year survival rate at < 50% for women younger than 65 years old and < 30% for women older than 65 years old [5]. Supported by the long-lasting benefits in a subset of patients, immune checkpoint blocking antibodies are currently being tested in OC patients and clinical responses have been documented in early trials [6, 7].

Enhancing the clinical efficacy of immune checkpoint blockade through combination therapies is a widely accepted hypothesis. As of September 2018, there were 2250 active clinical trials centred around the PD-1/L1 axis and nearly 60% of these trials were combinations [8]. Given the complexity of human tumour microenvironment, it is crucially important to develop pre-clinical tools to investigate the dynamic interplay between human cancer cells and the human immune system in response to immune checkpoint blockade.

Immunocompetent mice implanted with syngeneic tumour cells or genetically modified mice that develop spontaneous tumours are commonly used to assess efficacy of immunotherapeutic agents [9]. However, these models are limited in recapitulating the inherent heterogeneity in human cancer cells and the lymphocyte repertoire. To improve clinical relevance, humanised mice reconstituted with human immune cells on the immunodeficient background can be employed as an alternative [10]. Nonetheless, it is costly and time-consuming to construct these models, which could reduce their broad application. Using in vitro co-culture assays, we and others have utilised chimeric antigen receptors (CARs)-engineered human CD8+ T cells to study their killing mechanisms of human OC cells [11–13]. Although the engineered CD8+ T cells are highly potent in killing tumour cells, the requirement of pre-treatment with high-dose immune stimulating cytokines may alter the endogenous sensitivity to PD-1/L1-mediated immune suppression.

Inspired by recent studies where alloreactive T cells from healthy donors can recognise cancer-associated antigens [14, 15], we have established a tumour-immune co-culture system (TICS) with versatile functional endpoints to model the interplay between human cancer cells and unsorted primary human lymphocytes. TICS is built on the principles of an allogeneic response but has been specifically optimised to quantify activation of multiple human lymphocyte subsets and subsequent cancer cell killing upon PD-1/L1 blockade. Although we have previously reported the differential expression pattern and prognostic value of PD-L1 on human myeloid cells isolated from ovarian cancer patients [16], removal of monocytes from TICS is necessary to demonstrate the effects of immune checkpoint blocking antibody and to reduce donor-to-donor variability.

## Materials and methods

### Human cancer cell lines and primary human epithelial ovarian cancer (EOC) samples

Human breast cancer cell line MDA-MB-231 and human ovarian cancer cell lines OVCAR3, OVCAR4, OVCAR8, PEO1, PEO4, PEA1, PEA2, Ovsaho, Kuramochi and SKOV3 were maintained in IMDM medium (Thermo Fisher Scientific, Massachusetts, USA) supplemented with 10% heat-inactivated FBS and 1% PenStrep (Sigma-Aldrich, Poole, UK), referred as ‘assay medium’ below. Green fluorescent protein (GFP)-expressing MDA-MB-231 cell line was purchased from AntiCancer Inc., and maintained as above with the addition of 1 mg/ml G418 (Thermo Fisher Scientific, Massachusetts, USA). All cell lines have been authenticated by Eurofins Genomics (Ebersberg, Germany).

In experiments using primary EOC patient samples, ascites were obtained from treatment naïve patients with written consents in accordance with approved ethical permission by the West London Research Ethics Committee (Reference 12/WA/0196). Primary EOC tumour cells were isolated from ascites by gradient centrifugation with Ficoll-Paque Plus (GE Healthcare, Chalfont St. Giles, UK) and maintained in RPMI-1640 media (Sigma-Aldrich, Poole, UK) with 20% FBS (Sigma-Aldrich, Poole, UK), l-glutamine 200 mM, penicillin 10,000 units, streptomycin 10 mg/mL solution (Sigma-Aldrich, Poole, UK), 34 ng/ml insulin (Sigma-Aldrich, Poole, UK) and 2.2 mM sodium pyruvate (Sigma-Aldrich, Poole, UK). All cells were cultured at 37 °C with 5% CO_2_.

### Preparation of healthy donor primary human lymphocytes

Leukocyte cones were obtained from anonymous healthy blood donors, in accordance with the Human Tissue Act (NHS Blood Transfusion Unit, approval number M153). In order to isolate primary human peripheral blood mononuclear cells (PBMCs), blood products were first diluted in 50 ml PBS and laid carefully on top of 10 ml Lymphoprep reagent (StemCell Technology, Vancouver, Canada). The tubes were then centrifuged at 800 g for 20 min with the brakes off. Next, PBMCs were harvested from the interface above the Lymphoprep and washed twice in 50 ml PBS. In some cases, the cells were incubated in 5 ml ACK lysis buffer (Thermo Fisher Scientific, Massachusetts, USA) at room temperature for 3 min to remove the remaining red blood cells. Primary human monocytes were then depleted using EasySep CD14+ selection kit II (StemCell Technology, Vancouver, Canada) following the manufacturer’s instructions. The resulted primary lymphocytes were then resuspended in 3 ml PBS containing 1.5 μM CellTrace CFSE or CellTrace Violet Cell Proliferation Dye (Thermo Fisher Scientific, Massachusetts, USA), incubated at room temperature for 5 min and washed twice with 10 ml PBS. The ‘assay-ready’ lymphocytes were frozen at up to 100 million cells per vial and stored in liquid nitrogen tanks.

### Tumour-immune co-culture system (TICS)

To set up the TICS assay, human cancer lines or primary ascetic cells were seeded at indicated concentrations in 100 μl assay medium in a 96-well flat bottom plate and incubated at 37° overnight to allow cell adherence. On the same day, ‘assay-ready’ primary human lymphocytes were quickly thawed at 37 °C in a water bath, washed twice in 15 ml PBS, resuspended in assay medium and incubated at 37 °C with 5% CO_2_ overnight. Next, lymphocytes were resuspended at 3 million cells per ml and added to the wells containing tumour cells in 100 μl assay medium. To block the human PD-1/PD-L1 pathway, a human IgG4 isotype control (BioLegend, California, USA) or nivolumab (Bristol-Myers Squibb, New York, USA), a human IgG1 isotype control (NIP228, AstraZeneca, Cambridge, UK), a PD-1 blocking antibody (clone LO115, AstraZeneca, Cambridge, UK) or durvalumab (MEDI4736, AstraZeneca, Cambridge, UK) were added at 10 μg/ml. Activation of different immune cell subsets was analysed by flow cytometry on day 6, and soluble IFNγ levels in the supernatants were analysed by Human IFNγ MAX Deluxe ELISA kit (BioLegend, California, USA).

For real-time monitoring of tumour cell killing, TICS assay was set up in the presence of 1 μM green caspase-3/7 cell apoptosis reagent (Essen Bioscience/Sartorius, Göttingen, Germany) and imaged using an IncuCyte ZOOM instrument.

### Flow cytometry

All antibodies used for flow cytometry are summarised in Table S1. Briefly, cells were harvested and transferred to a 96-well v-bottom plate and then centrifuged at 800 g for 3 min. Cell pellets were washed twice in 200 μl PBS and resuspended in 20 μl PBS containing detection antibodies or cell viability dye. After a 30 min incubation at 4°, the cells were washed twice with 200 μl PBS and fixed in 1% paraformaldehyde. For CA-125 staining, following primary antibody incubation, cells were washed twice with 200 μl PBS and then incubated for 30 min with a goat anti-mouse PE-conjugated secondary antibody, prior to washing and fixing. BD LSRFortessa or BD FACSCanto II instruments (Becton Dickinson Immunocytometry Systems, California, USA) were used to acquire the data, and the results were analysed using FlowJo software version 6.4.7 (Tree Star Inc, Oregon, USA).

### siRNA knockdown and pharmacological inhibition

For knockdown experiments, siRNA targeting IFNγR (catalog number L-011057-00-0005), PD-L1 (catalog number L-015836-01-0005) or a non-specific siRNA control (catalog number D-001810-10-05) (GE Healthcare Dharmacon, Inc., Colorado, USA) were used. Tumour cells were seeded onto a six-well plate at a density of 2 × 10^5^ cells per well and cultured for 24 h. Before transfection, the cell culture medium was replaced with antibiotic-free medium. For each well to be transfected, the appropriate siRNA was diluted in Opti-MEM I Medium and complexed with Lipofectamine RNAiMAX (Thermo Fisher Scientific, Massachusetts, USA), following the manufacturer’s instructions, at a final siRNA concentration of 20 nM. After 24 h, the cells were trypsinised and seeded for TICS assay as described above.

The DNA methyltransferase inhibitor guadecitabine (SGI-110) was obtained from Astex Pharmaceuticals and reconstituted in its clinical diluent (65% propylene glycol, 25% glycerine, 10% dehydrated ethanol). Tumour cell lines were treated with the drug or vehicle at the indicated concentrations on day 1 and day 3. Cells were trypsinised and stained, as described above, for the quantification of surface expression of HLA-ABC and PD-L1 by flow cytometry on day 8. On the same day, to evaluate the effects of SGI-110 in TICS, vehicle or compound-treated cells were co-cultured with primary human lymphocytes as described above. In some experiments, blocking antibodies against antigen presentation molecules human HLA-ABC (clone W6/32, BioLegend) or HLA-DR (clone L243, BioLegend) were included.

### Statistical analysis

All results were summarised and analysed using a Graphpad Prism software. Appropriate statistical analyses were performed using paired Student *T* tests or two-way ANOVA as indicated in the figure legends.

## Results

### Immune checkpoint blockade enhances human cancer cell killing mediated by primary human lymphocytes

**Fig. 1:**
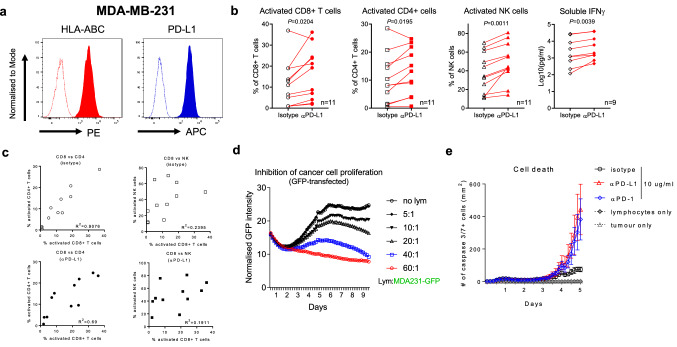
Immune checkpoint blockade enhances tumour cell killing mediated by primary human lymphocytes

In order to investigate the biological mechanisms of the PD-1/PD-L1 axis during human tumour–lymphocyte interaction, we have established and optimised a tumour-immune co-culture system, where unsorted healthy donor-derived primary human lymphocytes were co-cultured with human cancer cell lines (Figure S1a). The primary lymphocytes were labelled with a fluorescent dye, which shows reduced intensity as the cells proliferate. Surface markers CD38 and CD56 were used in combination with the cell trace reagents to identify activated T cells or NK cells in the proliferating cell subsets, respectively. Monocytes were depleted prior to the assay to improve reproducibility and reduce donor-to-donor variability. Utilising the mixed lymphocyte population allowed simultaneous analyses of activation and proliferation of CD4+ T cells, CD8+ T cells and NK cells as well as detection of immune modulatory factors released into the supernatant (Figure S1b). We first characterised the effects of PD-1 or PD-L1 blockade in the TICS using a human breast cancer cell line MDA-MB-231, due to its high expression of PD-L1 and HLA-ABC on the cell surface (Fig. 1a). As shown in Figure S2a, the magnitude of αPD-1-driven enhancement in lymphocyte activation and IFNγ levels was dictated by the cancer cell-to-lymphocyte ratio. Treatment with a Food and Drug Administration (FDA)-approved PD-L1 blocking antibody, durvalumab, resulted in significantly increased activation of CD8+ T cells (*p* = 0.0204), CD4+ T cells (*p* = 0.0195) and NK cells (*p* = 0.0011), as well as increased levels of soluble IFNγ (*p* = 0.0039, Fig. 1b) in 11 lymphocyte donors. Similarly, PD-1 blockade also increased activation of CD8+ T cells using multiple donor lymphocytes (Figure S2b). Interruption of antigen presentation machinery with blocking antibodies against HLA-ABC or HLA-DR abolished the activation of CD8+ or CD4+ T cells upon PD-L1 blockade, respectively (Figure S2c). Of note, there was a strong correlation between CD8+ and CD4+ T cell activation levels among donors in the isotype-treated groups (*R*^2 ^= 0.9075) as well as in the αPD-L1-treated group (*R*^2 ^= 0.69, Fig. 1c). In contrast, activation of NK cells showed weak correlation with CD8+ T cells (Fig. 1c) or CD4+ T cells (Figure S2d), indicating the non-overlapping regulation of different lymphocyte subsets during cancer–immune interaction.

To demonstrate the direct link between lymphocyte activation and cancer cell death in real-time, we have employed an IncuCyte live imaging platform. *In vitro* proliferation of the GFP-transfected human MDA-MB-231 cells was efficiently controlled by primary human lymphocytes in a dose-dependent manner (Fig. 1d). Using a fluorescent dye that detects active caspase 3/7 during cell death, we have confirmed that this inhibition was due to apoptosis of cancer cells mediated by activated primary lymphocytes rather than non-specific factors such as nutrient deprivation (Figure S3a). Indeed, either blocking PD-1 or PD-L1 enhanced cancer cell death in the presence of primary human lymphocytes. The effects were most evident after 3 days (Fig. 1e, Movie 1 and 2). Although the absolute counts of apoptotic cancer cells differ among lymphocyte donors in the TICS, the relative increases induced by an anti-PD-1 blocking antibody were highly consistent, as compared to the controls (Figure S3b).

### Human ovarian cancer (OC) cell lines are responsive to nivolumab in TICS

**Fig. 2:**
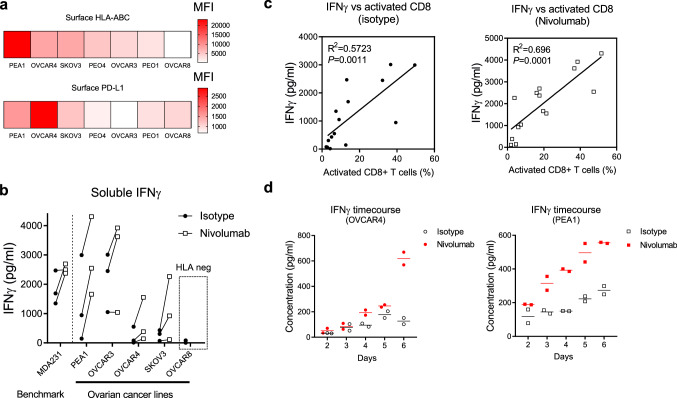
Human ovarian cancer cell lines are responsive to nivolumab in TICS

Because nivolumab has demonstrated disease control in a small cohort of OC patients [17], we sought to measure the lymphocyte activation primed by human OC cell lines in response to nivolumab in TICS. As shown in Fig. 2a, established human OC cells expressed varying levels of surface HLA-ABC and PD-L1. Using 3 lymphocyte donors in TICS, we have demonstrated the differential baseline release of immune stimulatory cytokine IFNγ as well as magnitude of response to nivolumab with selected OC cell lines (Fig. 2b). The MDA-MB-231 cancer cell line was included as a benchmark control. Of note, the HLA-ABC negative cell line OVCAR8 failed to elicit robust IFNγ release in TICS (Fig. 2b) and prime CD8+ T cell activation (data not shown), highlighting the necessity of TCR-HLA engagement for cancer cell recognition. Importantly, we have demonstrated strong correlations between IFNγ levels and activation of CD8+ T cells across lymphocyte donors and OC cell lines, both at the baseline (*R*^2 ^= 0.5723) and in presence of nivolumab (*R*^2 ^= 0.696) (Fig. 2c). In order to assess the dynamics of IFNγ release in TICS, we performed time-course measurements of IFNγ using OVCAR4 and PEA1 cell lines. Nivolumab-induced enhancement of IFNγ levels was detectable on day 3 in the PEA1 co-culture and was maximal on day 6. In contrast, nivolumab-induced IFNγ was not detected until day 6 in the OVCAR4 co-culture (Fig. 2d). Based on these data, we decided to use soluble IFNγ on day 6 as the readout for further experimental analysis.

### HLA and PD-L1 regulate cancer-driven lymphocyte activation

**Fig. 3:**
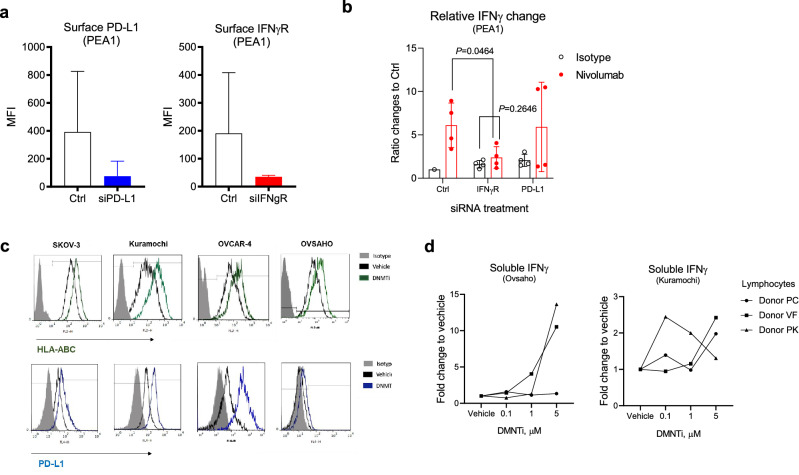
HLA and PD-L1 regulate cancer-driven lymphocyte activation

PD-L1 expression and defects in the IFNγ pathway have been reported to influence the efficacy of immune checkpoint blockade. To demonstrate the functional impact of the PD-L1 and IFNγ pathways, we used siRNA to knockdown PD-L1 and IFNγ receptor in PEA1 cells (Fig. 3a). To normalise the donor-to-donor variability of IFNγ concentrations at baseline, all values are shown as relative changes to the condition where cells were treated with the control siRNA and control isotype antibody. As expected, PD-L1 knockdown resulted in a 2.06-fold increase in baseline IFNγ release (2.06 ± 0.06, *n *= 4, Fig. 3b). IFNγ receptor siRNA did not clearly change the baseline IFNγ release in TICS (1.60 ± 0.43, *n *= 4, Fig. 3b). Moreover, nivolumab treatment substantially increased IFNγ release in cells treated with the control siRNA, but this effect was abolished when IFNγ receptor was silenced (*p* = 0.0464, *n *= 4).

Given the functional relevance of HLA-ABC and PD-L1 during the establishment of immune synapses, we hypothesised that enhanced expression of these molecules could improve lymphocyte activation in TICS. DNA methyltransferase inhibitors (DNMTi) have been reported to increase T cell-mediated cancer cell killing; one of the proposed mechanisms is through upregulation of MHC-I molecules [18]. Indeed, SGI-110, a DNMT inhibitor, upregulated surface expression of HLA-ABC and PD-L1 on four human ovarian cancer cell lines (Fig. 3c). As a result, ovarian cancer cell lines Ovsaho and Kuramochi, pre-treated with DNMTi, induced increased release of IFNγ in TICS (Fig. 3d).

### Reduced HLA expression in platinum-resistant human ovarian cancer cells limits PD-1 blockade

**Fig. 4:**
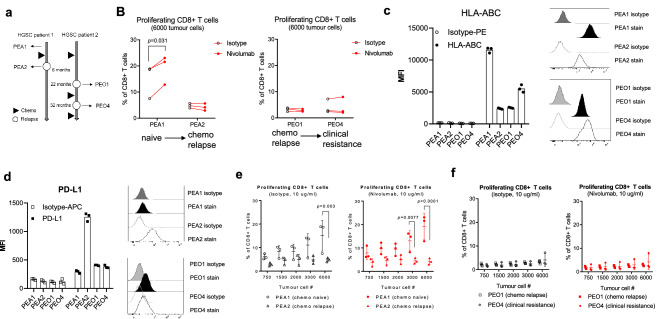
Reduced HLA expression in platinum-resistant human OC cells limits PD-1 blockade

Currently, the standard of care therapy for patients with high-grade serous carcinoma (HGSC) is platinum or taxane-based chemotherapy. In order to explore the impact of acquired chemotherapy resistance on baseline lymphocyte activation and response to PD-1 blockade, we have tested in the TICS two HGSC cell line pairs that were established from ascites at different treatment stages (Fig. 4a). The PEA1/PEA2 pair was established from a patient prior to chemotherapy (naïve) and at the point of relapse after 6 months (chemo relapse), respectively [19, 20]. The PEO1 cell line was established from a patient following treatment and chemo-sensitive relapse, while the PEO4 cell line was established following the development of clinical resistance [20, 21].

**Fig. 5:**
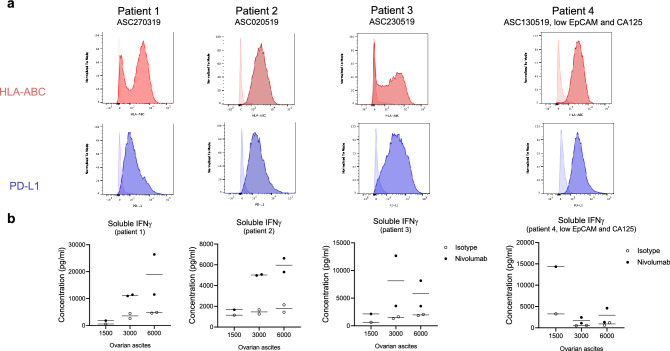
Treatment naïve primary ovarian cancer cells respond to PD-1 blockade in TICS

In TICS, treatment naïve PEA1 cells induced activation of CD8+ T cells, which was significantly enhanced by nivolumab (*p* = 0.031, Fig. 5b). Strikingly, the paired, chemotherapy-relapsed, PEA2 cell line did not induce strong T cell activation and failed to respond to PD-1 blockade (Fig. 3b). The reduced cancer-driven lymphocyte activation of PEA2 cells was confirmed using a clinically approved PD-L1 blocking antibody (data not shown). This lack of immune activation and response to PD-1 blockade in chemotherapy-resistant ovarian cancer cells were confirmed in the PEO1 and PEO4 cell lines, both of which were established after the patient has relapsed from chemotherapy (Fig. 4b). Measuring cell surface expression levels of HLA-ABC and PD-L1 in the pairs showed that expression of neither protein can explain anti-PD-1 response levels. However, PEA1 showed the highest HLA-ABC expression among the cell lines (Fig. 4c, d), which could in part contribute to its higher level of immune activation.

To rule out that these functional differences on immune activation are skewed by cancer-to-lymphocyte ratios, we have co-cultured a fixed number of primary human lymphocytes with increasing numbers of cancer cells. While tumour cell numbers played a role in the activation of CD8+ T cells co-cultured with the PEA1 cell line, the lack of immune response to PD-1 blockade in PEA2 cells (Fig. 4d) and the PEO1/PEO4 (Fig. 4e) pair were consistently seen in all ratios tested.

### Treatment naïve primary ovarian cancer cells respond to PD-1 blockade in TICS

To confirm that treatment naïve primary OC cells could elicit lymphocyte activation in response to PD-1 blockade, we have obtained purified cancer cells from patient ascites. After gradient centrifugation, three out of four samples were enriched for cells expressing ovarian cancer cell markers EpCAM or CA-125 (Figure S4). In contrast, only low levels of macrophages were present (5.31 ± 2.36%) and less than 1% monocytes or T cells (data not shown) were detected (Figure S4). Next, we confirmed the expression of surface HLA-ABC and PD-L1 on the primary OC cells. Regardless of cell composition, all four samples showed detectable levels of HLA-ABC and PD-L1 (Fig. 5a). When tested in TICS, cancer cells isolated from patients 1, 2 and 3 induced detectable levels of soluble IFNγ, which were further enhanced after nivolumab treatment (Fig. 5b). Notably, cells isolated from patient 4 lacked EpCAM or CA-125 positive cancer cells, which did not show consistent enhancement of IFNγ in response to nivolumab (Fig. 5b).

## Discussion

In this study, we have utilised a tumour-immune co-culture system to investigate human ovarian cancer intrinsic mechanisms in controlling primary lymphocyte activation and response to immune checkpoint blockade. Our data suggest a ‘cross resistance’ model, where functional alterations of cancer cells, such as the loss of HLA class I molecule, in chemotherapy-resistant OC cancer cells may hamper the efficacy of immune checkpoint blocking antibodies.

TICS is a feasible platform with multiple functional endpoints that are tailored for the PD-1/L1 blocking agents. It offers a pre-clinical tool to bridge *in vivo* syngeneic, humanised mouse models and *ex vivo* functional assays using patient-derived cells. We have demonstrated the robust activation of human CD8+, CD4+ T cells and NK cells in response to PD-1/L1 blockade. This enhanced immune activation results to reduced cancer cell proliferation due to immune-mediated cancer cell killing. Of note, activation of CD8+ T cells correlates strongly with CD4+ T cells and the release of IFNγ but does not show a strong correlation with NK cell activation. In line with a recent study, this supports the indispensable and non-redundant role of NK cells in cancer immunotherapy [22] and could be employed to reveal functional effects of NK cell stimulating therapies such as anti-NKG2A mAb [23] or interleukin 15 [24]. In TICS, live cell imaging reveals that cancer cell lysis by primary human lymphocytes in response to PD-1/L1 blocking antibodies initiates after 3 days. This is in contrast to genetic-engineered or cytokine-activated T cells [11–13] or NK cells [24], which readily mediate cancer cell killing within hours in vitro, and which therefore may not be as sensitive to inhibition through immune checkpoint molecules. Moreover, the functional and mechanistic involvement of additional lymphocyte subsets upon PD-1/L1 blockade, such as B cells, gamma-delta T cells, and invariant NK cells, can be dissected in TICS. Monocytes are removed from the TICS to improve technical reproducibility among donors; therefore, the assay is best suited to interrogate the impact of oncogenic pathways on PD-1/L1 blockade.

The immune modulatory effects of chemotherapy are well documented. For example, chemotherapy could deplete cell types with immunosuppressive functions, activate stimulatory functions in antigen presenting cells and induce immunogenic cell death, apoptosis or autophagy in cancer cells [11, 25]. In pre-clinical models of ovarian cancers, cisplatin elicits anti-tumour immune responses through activation of the cGAS/STING pathway [26]. In cancer patients, combination of chemotherapy and immune checkpoint blocking antibodies has shown encouraging efficacy in treatment-naïve patients in randomised late-stage clinical trials, which lead to two FDA approvals in extensive stage small cell lung cancer (IMpower133, Roche) [27] and triple-negative breast cancer (IMpassion130, Roche) [28], respectively. Emerging data from phase 3 clinical trials also demonstrate prolonged progression free survival in small cell lung cancer patients treated with chemotherapy and immunotherapy combinations (Caspian, AstraZeneca; Keynote-604, Merck). These data support the hypothesis that the induction of durable anti-tumour immunity is more achievable in the first-line setting or early stage malignancies.

Although most OC patients respond to the initial platinum-based chemotherapy, many relapse and acquire resistance during the treatment cycles. As such, it is timely and crucially important to investigate how chemotherapy resistance influences the response to immune checkpoint blockade therapy.

Here, we have utilised established cell line pairs, PEA1/2 and PEO1/4, to investigate the impact of chemotherapy resistance developed at different treatment cycles on the response to immunotherapy in ovarian cancers. Upon treatment with platinum-based chemotherapeutics *in vitro*, growth inhibition is seen in all four cell lines (PEA1/PEA2 and PEO1/PEO4) [29], but PEA1 and PEO1 cell lines are reportedly more sensitive to cisplatin treatment as compared to PEA2 and PEO4 [21]. At the molecular level, PEA1 cells do not harbour distinct oncogenic mutations as compared to the other three cell lines and none of the cell lines shows aberrations in microsatellite instability (MSI) or BRCA [30]. However, only the PEA1 cell line, derived from a chemotherapy naïve patient, elicits strong activation of primary human CD8+ T cells in the TICS and demonstrates a statistically significant increase upon PD-1 blockade. This is in line with a recent report, where chemotherapy naïve PEA1 and PEO14 show higher CD103 expression on human T cell activated with a CD3 agonistic antibody as compared to their chemo-resistant counterparts PEA2 and PEO23, respectively [31].

We propose that the superior activation of human lymphocytes primed by PEA1 cells is largely due to the high expression of HLA class I molecules as compared to the chemotherapy-resistant cell lines. This is further supported by the observation that ascites containing cancer cells from treatment naïve OC patients expressed HLA class I and demonstrate enhanced immune activation in the presence of nivolumab. It is textbook knowledge that HLA molecules can be induced by interferon-γ derived from activated immune cells. Recently, loss of the JAK/STAT signalling downstream of IFN receptors has been observed to confer resistance to PD-1 blockade therapy in human melanoma patients [32, 33]. Indeed, knocking down IFNγ receptor by siRNA on PEA1 OC cells compromises the effect of nivolumab in TICS. Silencing of the IFNγ receptor does not cause substantial reduction in immune cell activation at the baseline, possibly due to the low levels of IFNγ present in the co-culture. However, it remains unclear whether the down-regulation of HLA proteins is a direct effect of chemotherapeutics on OC cancer cells or a result of selection pressure from activated immune responses during treatment.

Recently, targeting epigenetic regulators has been proposed as a novel cancer treatment strategy and a potential approach to enhance anti-tumour immune responses [34]. One possible mechanism of action is the enhanced recognition of cancer cells by CD8+ T cells through up-regulation of HLA class I molecules and tumour-associated antigens on cancer cells [18]. We have confirmed that a DNMT inhibitor is able to enhance expression of HLA class I molecule and PD-L1 on human OC cell lines. As a result, pre-treatment of human OC cell lines with this inhibitor increases the activation of immune cells in TICS. However, because epigenetic regulators may alter the expression levels of a broad range of proteins, further work is warranted to dissect the immune regulatory roles of these compounds.

Immune checkpoint blockade therapy has proven efficacy in multiple cancer types and is currently being evaluated in human OC patients. Our study suggests that ‘cross resistance’ caused by the down-regulation of HLA class I molecules on chemotherapy-resistant OC cancer cells may limit efficient activation of the adaptive immune system. As such, it is reasonable to speculate that immunotherapy may be more effective as a first-line therapy or in combination with agents that can enhance expression of HLA molecules in OC patients.

### Electronic supplementary material

Below is the link to the electronic supplementary material.

Supplementary material 1 (WMV 26388 kb)

Supplementary material 2 (WMV 26395 kb)

Supplementary material 3 (PPTX 6582 kb)

Supplementary material 4 (DOCX 14 kb)

## Abbreviations

DNMTi: DNA methyltransferase inhibitor
GFP: Green fluorescent protein
MSI: Microsatellite instability
OC: Ovarian cancer
PBMCs: Peripheral blood mononuclear cells
PD-1: Programmed cell death protein 1
PD-L1: Programmed death-ligand 1
TICS: Tumour-immune co-culture system
TMB: Tumour mutational burden
