# Supplementary material for: Human ovarian cancer intrinsic mechanisms regulate lymphocyte activation in response to immune checkpoint blockade
Source: Cancer Immunol Immunother. 2020 Mar 21;69(8):1391–401. doi: 10.1007/s00262-020-02544-5 (PMC7347689; doi:10.1007/s00262-020-02544-5)
Supplement: Supplementary file 3 — Supplementary material 3 (PPTX 6582 kb) [file 262_2020_2544_MOESM3_ESM.pptx]

## Slide 1
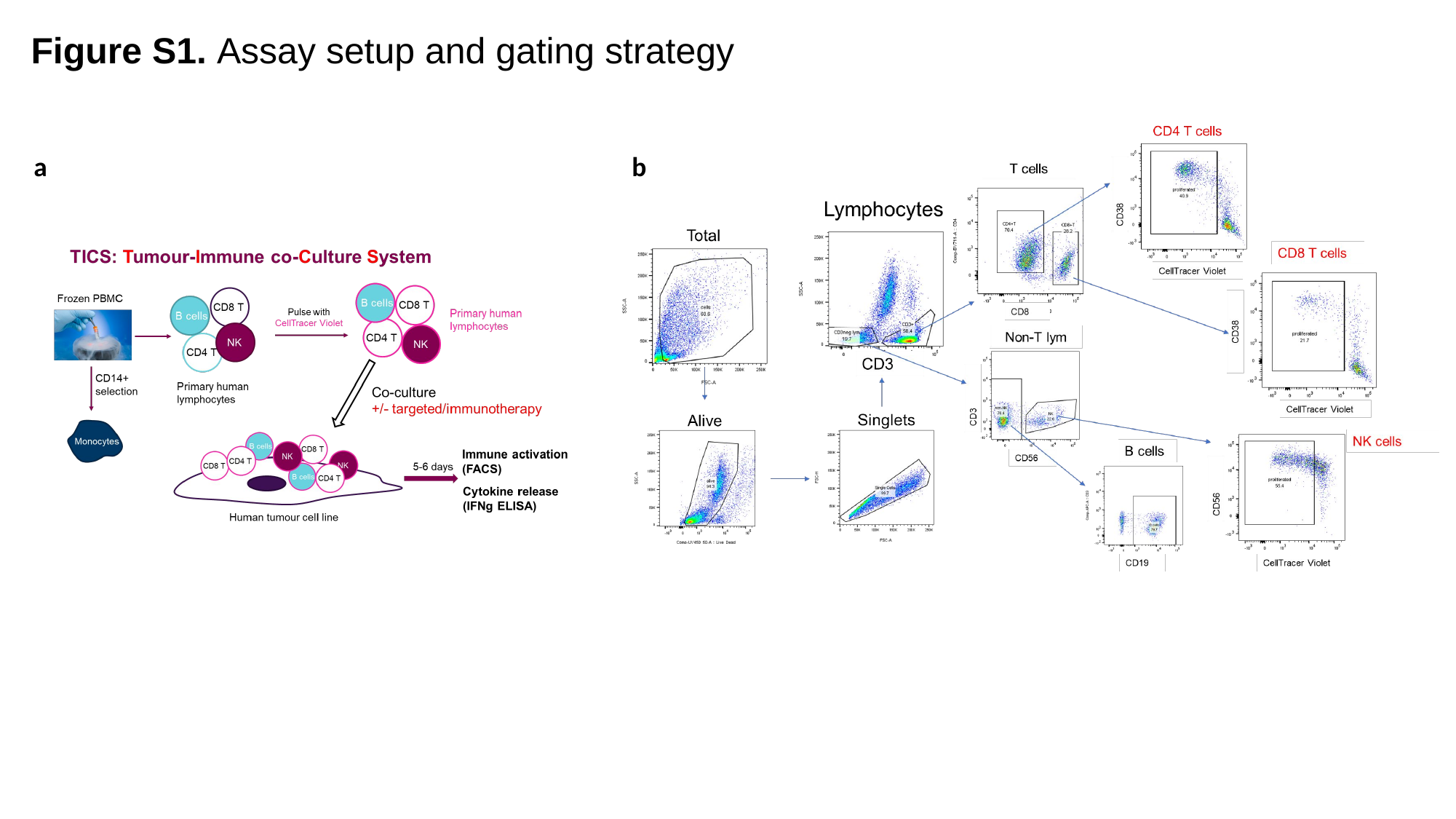

# Figure S1. Assay setup and gating strategy
b
a

## Slide 2
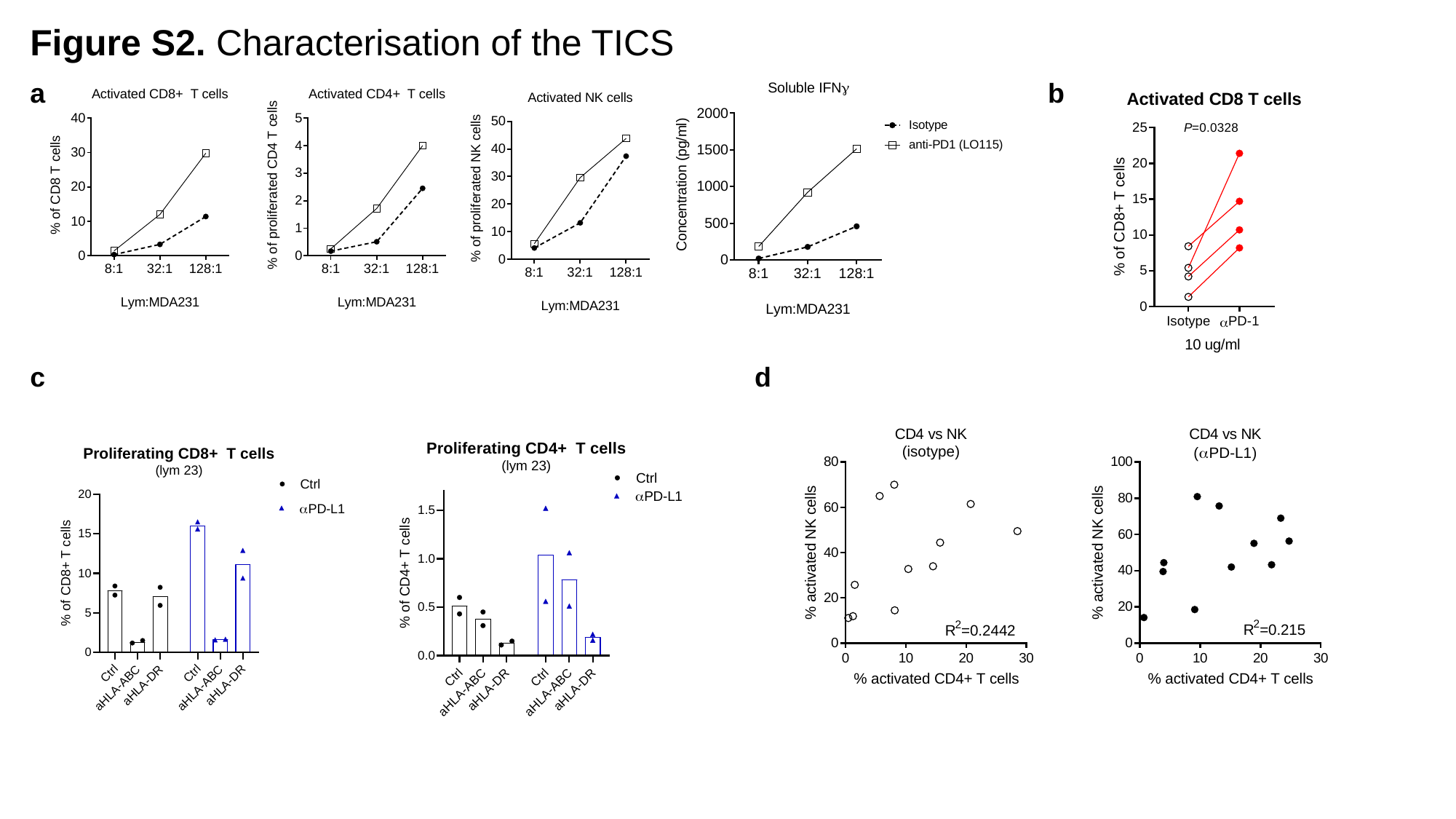

# Figure S2. Characterisation of the TICS
a
b
c
d

## Slide 3
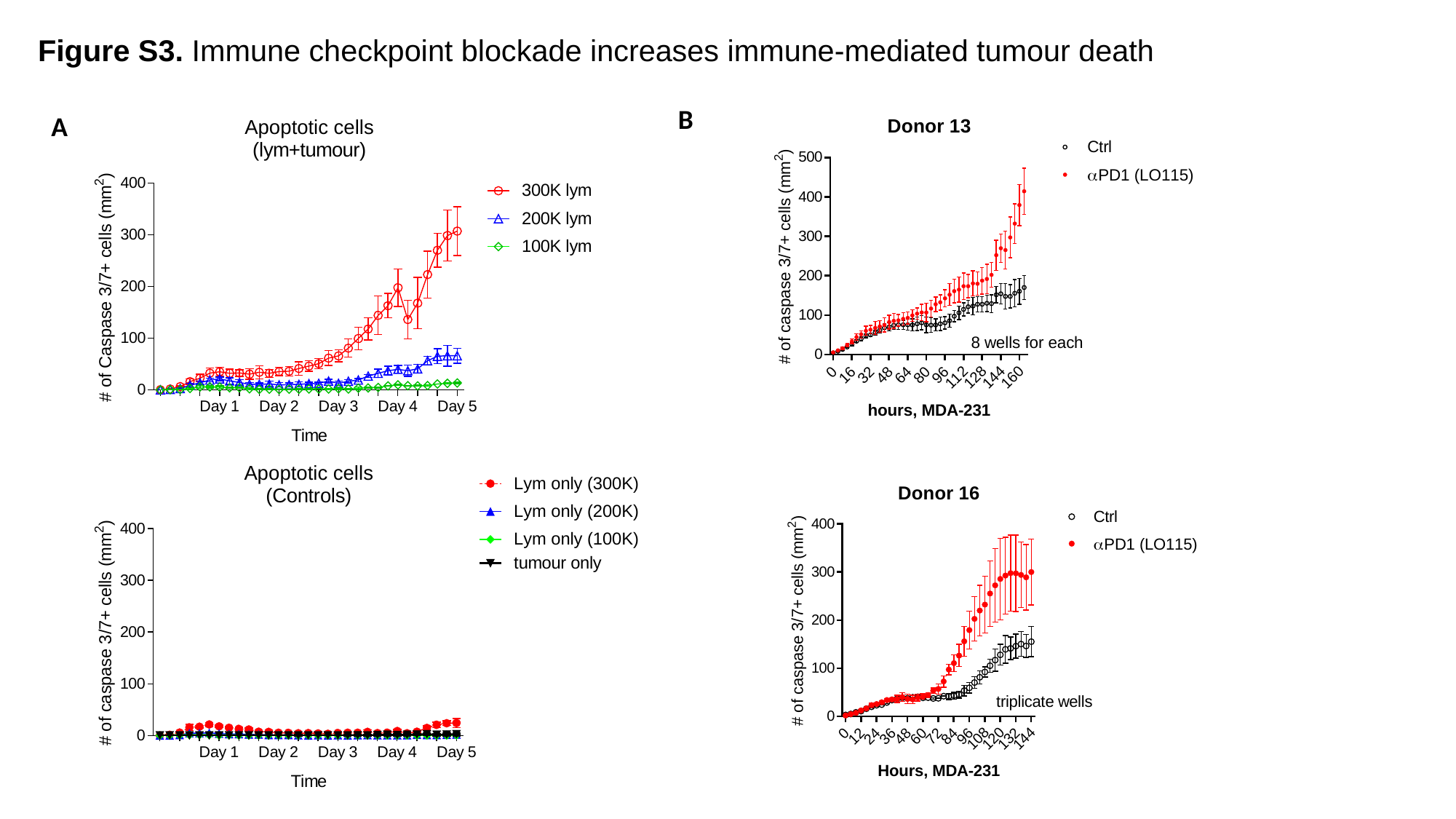

Figure S3. Immune checkpoint blockade increases immune-mediated tumour death
B
A

## Slide 4
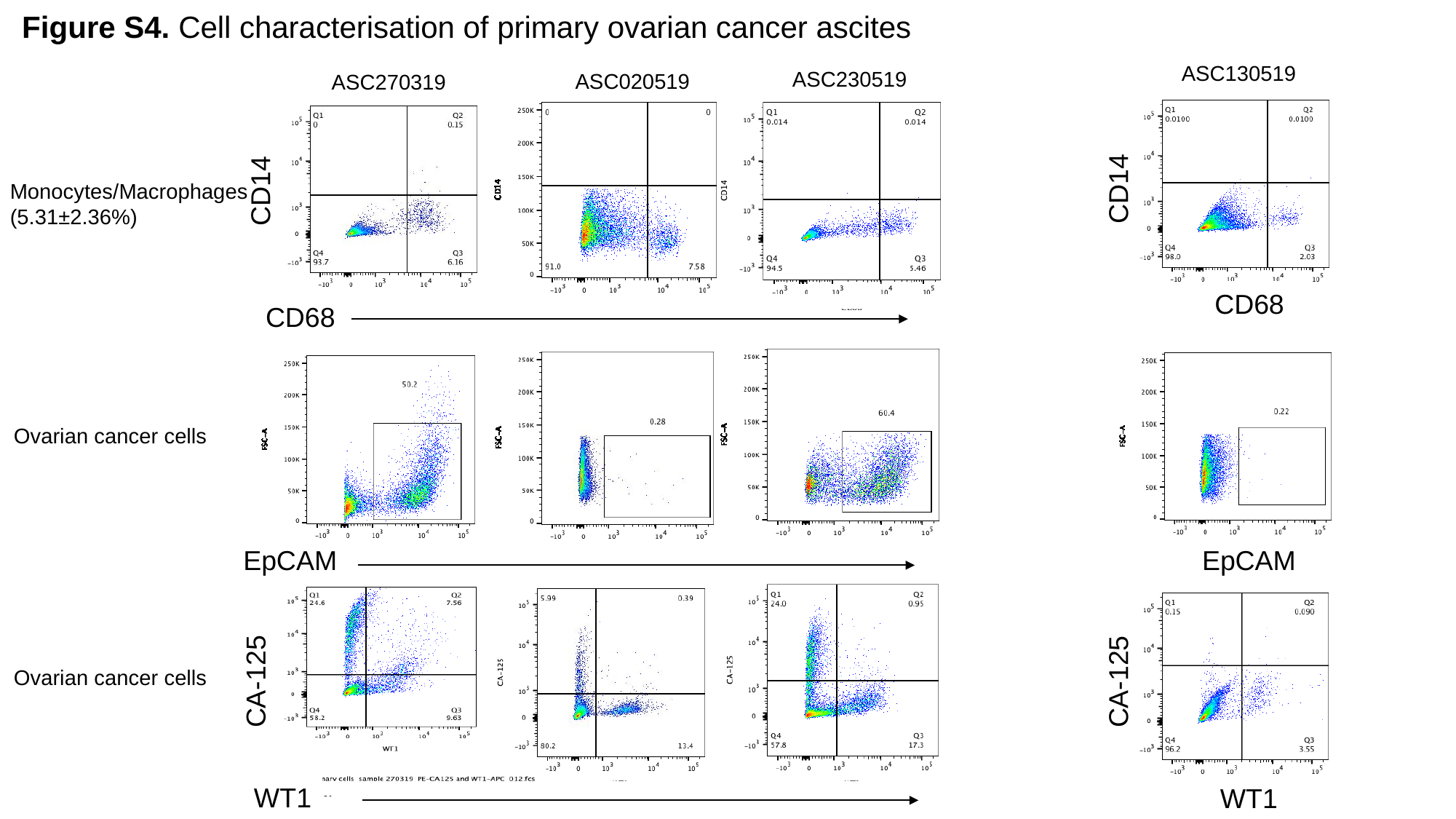

Figure S4. Cell characterisation of primary ovarian cancer ascites
ASC130519
ASC230519
ASC020519
ASC270319
CD14
CD68
CD14
Monocytes/Macrophages
(5.31±2.36%)
CD68
EpCAM
Ovarian cancer cells
EpCAM
CA-125
WT1
Ovarian cancer cells
CA-125
WT1
