# Supplementary material for: Human ovarian cancer intrinsic mechanisms regulate lymphocyte activation in response to immune checkpoint blockade
Source: Cancer Immunol Immunother. 2020 Mar 21;69(8):1391–401. doi: 10.1007/s00262-020-02544-5 (PMC7347689; doi:10.1007/s00262-020-02544-5)
Supplement: Supplementary file 4 — Supplementary material 4 (DOCX 14 kb) [file 262_2020_2544_MOESM4_ESM.docx]

**Supplemental table and figure legends**

| **Table S1. Reagents used for flow cytometric analysis** | | |
| --- | --- | --- |
| **Antigen** | **Fluorochrome** | **Provider/Catalog number** |
| CD3 | APC | Biolegend/317318 |
| CD4 | BV711 | Biolegend/317440 |
| CD8 | BV650 | Biolegend/301042 |
| CD8 | PerCP/Cyanine5.5 | Biolegend/301032 |
| CD56 | PE-Cy7 | Biolegend/318318 |
| CD38 | AlexaFluor 488 | Biolegend/303512 |
| CD19 | PE | Biolegend/302208 |
| Live/dead | UV450 | ThermoFisher Scientific/L23105 |
| CA-125 | / | Biolegend/666904 |
| WT1 | Alexa Fluor® 647 | Abcam/ ab202639 |
| EpCAM | PE | Invitrogen/12-9326-42 |
| HLA | PE | Invitrogen/12-9983-42 |
| PD-L1 | APC | Biolegend/329708 |
| Mouse IgG2a k | PE | Invitrogen/ 12-4724-81 |
| Mouse IgG1 k | PE | Invitrogen/ 12-4714-42 |
| Mouse IgG2b κ | APC | Biolegend/400322 |
| Goat anti-mouse secondary Ab | PE | Dako/R0480 |

**Figure S1. Assay setup and gating strategy.** a) Graphic illustration of the TICS. b) Gating strategy to analyse lymphocyte activation in TICS.

**Figure S2. Characterisation of the TICS.** a) A fixed number of primary human lymphocytes are co-cultured with decreasing numbers of human breast cancer cell line MDA-MB-231 in presence of an isotype control antibody or an αPD1 mAb (clone LO115, 10 μg/ml). The resulted activation of CD8+ T cells, CD4+ T cells and NK cells, as well as soluble IFNγ levels are measured. b) Effects of an isotype or αPD1 mAb (clone LO115, 10 μg/ml) on the activation of CD8+ T cells are shown in 4 independent lymphocyte donors, paired T test. c) Representative experiment to demonstrate the effects of blocking antibodies against human HLA-ABC and HLA-DR on the proliferation of CD8+ and CD4+ T cells in presence or absence of a PD-L1 blocking antibody. d) Correlations between the activation of CD4+ T cells and NK cells in presence of an isotype control antibody or an αPD-L1 antibody in TICS as a supplement to Figure 1c.

**Figure S3. Immune checkpoint blockade increases immune-mediated tumour death.** a) Human breast cancer line MDA-MB-231 cells and primary human lymphocytes are culture alone or together in presence of a fluorescent dye that stains for apoptotic marker active caspase 3/7. The cell death is monitored in real-time using Incucyte up to 5 days. b) Death of MDA-MB-231 in TICS is monitored in real-time using Incucyte in presence of an isotype or αPD1 mAb (clone LO115, 10 μg/ml) in 2 independent experiments. Data is presented as mean±SD.

**Figure S4. Cell characterisation of primary OC ascites.** OC cancer cells are enriched from 4 treatment naive primary ascites samples using gradient centrifugation, followed by characterisation using human monocyte maker CD14, human macrophage marker CD68 and OC markers EpCAM and CA-125 in FACS.

**Movie 1 and 2. Immune-mediated inhibition of cancer cell proliferation by PD-1/L1 blockade.** Primary human lymphocytes are co-cultured with a GFP-transfected human breast cancer line, MDA-MB-231, in presence of a PD-1 blocking antibody or the corresponding isotype control (10 µg/ml). Growth of cancer cells is measured in real-time using an Incucyte live-cell imaging instrument for 6 days.
